# Use of Acupuncture in an Infant with Restlessness and Agitation

**DOI:** 10.3390/medicines5020055

**Published:** 2018-06-13

**Authors:** Katharina Murg, Wolfgang Raith, Berndt Urlesberger

**Affiliations:** 1 Division of Neonatology, Department of Paediatrics and Adolescent Medicine, Medical University of Graz, Graz 8036, Austria; wolfgang.raith@klinikum-graz.at (W.R.); berndt.urlesberger@medunigraz.at (B.U.); 2 Research Group for Paediatric Traditional Chinese Medicine, TCM Research Centre Graz, Medical University of Graz, Graz 8036, Austria

**Keywords:** acupuncture therapy, baby, restlessness, agitation

## Abstract

Abstract: **Background:** We are reporting here about a 3-month-old boy with a history of failure to thrive, hypertrophic obstructive cardiomyopathy and neurological misbehaviour including hypotension in body muscles, who was found to have screaming attacks, agitation and restlessness. **Methods/Results:** Body and ear acupuncture was used both as supporting and integrative therapy to reduce the phases of restlessness and screaming and, simultaneously, the use of hypnotic drugs, as well as to improve the baby’s thriving. **Conclusions:** Our case has proved that standardised ear and body acupuncture applied by trained acupuncturist paediatricians is a helpful non-pharmacological treatment tool. While acupuncture is typically used in the outpatient setting, it can equally be used in the inpatient setting, as exemplified by the positive outcome of the presented case.

## 1. Introduction

In recent years, the interest in complementary and integrative therapies for children has increased significantly [1,2]. Traditional Chinese Medicine (TCM) has been reported as one of the most popular complementary treatments in children and neonates [3,4]. TCM includes (i) massage therapy (Tuina); (ii) moxibustion; and (iii) different kinds of acupressure and acupuncture [5,6]. The most common complementary applications for acupuncture include pain [7,8] and chemotherapy-induced nausea/vomiting [9]. To this day, only a few studies have investigated the effect of needle acupuncture in infants [10,11,12,13]. Even this limited available data suggests that acupuncture may be applied as a safe, non-pharmacological treatment option for the reduction of pain and agitation in term and preterm infants [14,15,16].

This case report illustrates the feasibility, safety, and efficacy of needle acupuncture in an infant with various medical dilemmas who was admitted to the intensive care unit.

## 2. Case Report

We are reporting here about a 3-month-old infant who revealed a history of swallowing problems, gastroesophageal reflux, failure to thrive, hypertrophic obstructive cardiomyopathy, neurological abnormalities, and screaming attacks ever since his birth. He was the second child of healthy, non-consanguineous parents, born in an uncomplicated vaginal delivery at 40 + 1 pregnancy weeks with an APGAR score of 9 at 1 min and a score of 10 at five and 10 min. Postnatal adaption was unremarkable, however, with an obvious macrosomia without the evidence of maternal gestational diabetes. During the first few weeks of his life, the baby boy showed poor thriving, and agitation, and at the age of three months, hospital admittance was recommended. Echocardiography showed hypertrophic obstructive cardiomyopathy. To rule out Sandifer, Costello syndrome, and Mb—pompe, genetic and metabolic investigations were performed but revealed no evidence of any congenital syndromes (until today). One day after admission, the patient showed signs of respiratory distress, phases of restlessness and agitation. The skin was pale; he was sweating and demonstrated ophistotonus. The heart rate increased intermittently up to 180/min while oxygen saturation decreased to 75% in ambient air conditions. The screaming attacks lasted for one hour, and calming the infant was almost impossible. The patient was subsequently transferred to the paediatric intensive care unit (ICU) where he received hypnotic drugs. Parenterally, Phenobarbital (5 mg/kg/day), Clonidine (10 µg/kg/day), Midazolam (0.5 mg/kg/day) and homeopathic suppositories were administered for 5–7 days, however, they had little impact. Additionally, submitter nutrition by gavage turned out to be necessary due to feeding problems and drinking weakness of the infant.

### Acupuncture Therapy

**Figure 1:**
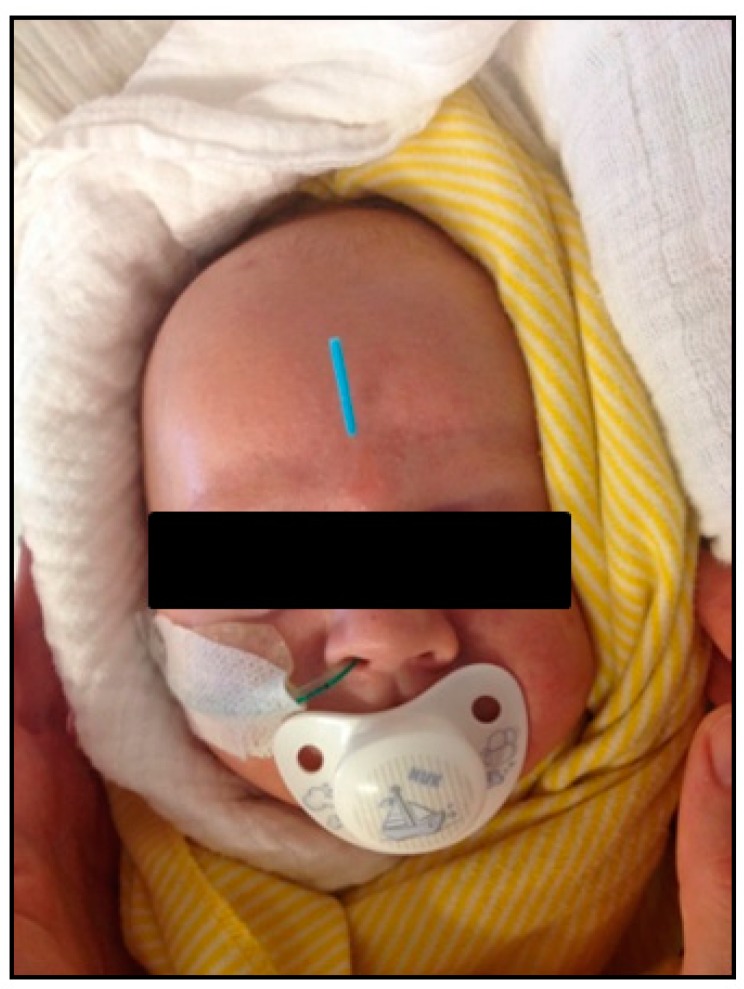
Sleeping baby with a needle positioned at Yin Tang.

Acupuncture was used as adjunctive therapy with the aim to reduce the phases of restlessness and screaming, and it was hoped to reduce the need for hypnotic drugs. Body and ear acupuncture were performed by two trained specialists. Sterile and thin disposable acupuncture needles (0.20 × 15 mm) were applied at acupuncture points LR 3, LI 4 and ST 36 on both sides of the body. The needle was inserted 2–4 mm deep into the baby’s skin and manipulated slightly until a certain sensation of resistance was felt and then left in place for approximately 10 to 20 s before withdrawal. When doing the Yin Tang point, the needle was left in place for approximately 10 to 15 min. Furthermore, ear acupuncture was performed at Shen Men with an indwelling needle (0.20 × 1.2 mm) lasting 24 h. A total of six sessions were applied, performed every other day. The skin response at the needle’s insertion points was noticeable. During the acupuncture sessions the patient was quiet, relaxed and would even fall asleep, especially after tapping the Yin Tang point. (Figure 1) Strikingly, both the frequency and duration of the infant’s screaming attacks and restlessness decreased after only one acupuncture treatment. The patient was found to be calmer, his handling was now easier and his body tension was found to be significantly reduced. All this was reported back to us by the nursing staff. As a result, the sedative medication dose was able to be reduced on a daily basis. After one week of acupuncture treatment, Clonidine and Midazolam were discontinued, and the patient was now able to drink infant formula via bottle feeding.

## 3. Methods

The acupuncture points are here described following the international nomenclature [17]. The treatment itself is described according to the current Standards for Reporting Interventions in Clinical Trials of Acupuncture (STRICTA) guidelines [18,19].

Yin Tang (“Point de Merveille”, EX-HN3) [20] located in the midpoint between the eyebrows was chosen due to its sedative and analgesic effects, in addition to its anxiolytic and stress-reducing impact [14,21,22].

Tai Chong (Liver 3, LR 3) located on the dorsum of the foot, in a depression distal to the junctions of the first and second metatarsal bones, was used as calming point to reduce the irritability, anger, insomnia and anxiety of the baby [23,24]. 

Additionally, we did the Hegu (Large Intestine 4, LI 4), situated on the dorsal surface of the hand between the first and second metacarpal bones, as a general pain point and to regulate the face and head areas. This point was also identified to minimise swelling and irritability [25,26].

Zusanli (Stomach 36, ST 36), located in the tibialis anterior muscle, 3 cun below ST 35 and one finger-breath lateral of the anterior border of the tibia was selected to reduce gastric symptoms such as dysphagia, vomiting, nausea and to activate the baby’s appetite [27,28]. 

Recognised as one of the most effective ear-acupuncture points, we used Shen Men (ear point no 55) situated at the apex of the triangular fossa. It was used to alleviate apprehension, fear, and anxiety, reduce addiction to sedative medication, and to regulate the sympathetic nervous system [29]. Comparable effects to ear acupuncture were demonstrated by Capone et al. after transcutaneous vagus nerve stimulation at an external acoustic meatus that produces an increased cortical inhibitory modulation of the right motor cortex [30].

Written informed consent was obtained from the baby’s mother.

## 4. Discussion

To establish this case report, we examined the use of acupuncture as a possible therapy to decrease and treat restlessness and agitation in a new-born infant in the paediatric intensive care unit (PICU). Generally, acupuncture can be performed both invasively (by needling or in the traditional manner) or non-invasively (e.g., using magnets, laser, acupressure) and has meanwhile been used extensively in adults as well as in children to treat myriads of problems [31].

Recently, there have been several publications evaluating the effects of needle acupuncture during minor painful procedures and on mortality and morbidity in neonates with hypoxic ischemic encephalopathy with promising results, i.e., that preterm infants respond very well indeed to needle acupuncture therapy [14,32]. 

At the moment, however, it is still unknown whether repeated needle stimulation may alter sensory processing and responses to subsequent painful stimuli in the same manner as heel sticks applied in infants [33]. 

Kundu et al. performed a retrospective review on infants (*n* = 12, aged 2 to 17 years, median, 4 years) who suffered an emergence delirium on a previous general anaesthetic and received acupuncture with a subsequent anaesthetic. Eighty-three-percent did not exhibit any symptoms of agitation, while 17% exhibited mild symptoms. Moreover, patients in the acupuncture group were found to cope with a smaller dosage of an intravenously administered anaesthetic when comparing their anaesthetic requirements before and after the intervention [34].

While causal links could not be concluded from the findings in our case report, it does seem more than likely that the acupuncture treatments contributed to the resolution of the symptoms; we therefore wish to discuss the acupuncture points used with respect to the existing scientific literature:

Ecevit et al. conducted a crossover study in 10 preterm infants who were randomised to routine care (breast milk, non-nutritive sucking) and acupuncture, or routine care alone. Each infant acted as their own control, and crossover occurred after one day, i.e., infants who received acupuncture on day 1 would receive routine care on day 2. Superficial needling acupuncture therapy was carried out for 30 min prior to heel prick at Yin Tang. The Neonatal Infant Pain Scale score, mean crying duration and post-procedural heart rate were all found to be significantly lower in infants who had received acupuncture [14]. In addition, it was revealed in a clinical study carried out by Tugcu et al. that SaO2 values increased and the pulse rate decreased significantly in infants on whom acupressure on Yin Tang had previously been applied [21]. Other studies specifically address the effect of acupuncture interventions on withdrawal and agitation in children. Wang et al. found that acupressure on Yin Tang reduced anxiety in children undergoing anaesthesia [20]. Due to these facts, we have meanwhile included this acupoint in our overall therapy concept. In the patient under discussion here, the heart rate remained low throughout the various acupuncture sessions. 

Our selection of acupoint LI4, one of the most commonly used acupuncture points in TCM, was owing to its well-established stimulation of microcirculatory and analgesic effects [35,36]. 

Reinthal et al. have investigated gastrointestinal symptoms of infantile colic in 913 infants after light needling in LI4. After treatment, the variables of inflated stomachs, drooling and regurgitation were found to have changed while the defecation frequency was found to be reduced. The parents also rated their perceived impressions of their children’s general changes of colic symptoms including crying behaviour ameliorated in as much as 76% of the cases [12]. Similar results have been reported by Lindgren et al. in newborns with infantile colic receiving acupuncture on LI4, who had shorter periods of crying due to better relaxation [11]. We ourselves have reported about an improved feeding behaviour. Another study by Usichenko et al. has shown that stimulation of LI4 in children reduces pain and autonomic distress [25].

ST36 was chosen since it is the main focus of the physical body found in Chinese literature. In addition, safety in the application of ST36 has been proven in several studies [10,37].

Gottschling et al. have explored the need of antiemetic therapy in 23 paediatric oncological patients receiving highly emetogenic chemotherapy for solid malignant tumours after supportive acupuncture treatment. Episodes of vomiting and requirement for antiemetic medication including 5-HT3-antagonist and Cortisone were found to be significantly lower in the acupuncture group as compared to the control group. The study showed that acupuncture of ST36 and LI4 (among others) added to pharmacotherapy was more effective in preventing chemotherapy-induced nausea and vomiting than antiemetic medication alone [9].

In particular, stimulation of acupoint LR3 activates several cortical and subcortical regions responsible for acute and chronic pain [23], and auricular acupuncture potentially modifies autonomic dysfunction by increasing parasympathetic activity and reduces sympathetic hyperactivity [38,39].

Observations of Wu et al. have presented evidence of the analgesic efficacy of acupuncture at LR3 using functional magnetic resonance imaging (fMRI). During the emergence of needle sensation at LR3, the thalamus region, being a component in the pain management network, was deactivated. Findings in the fMRI may show a correlation between functional brain areas and the regulatory effects of acupuncture at LR3 [23].

Another primary reason that complementary methods have turned out highly beneficial in inpatient paediatric settings can be seen from the infants’ relaxation, especially in agitated babies with NAS [40]. It has also been shown that the combination of ear and body acupuncture is very successful [41]. In a study carried out in neonates with NAS, the addition of auricular acupressure beads did not result in a different clinical course, though there was a suggestive trend towards less pharmacological support needed in the acupressure group [42]. In their publications, Gentry et al. and Raith et al. have repeatedly described the positive effects on improved sleeping behaviour due to a better relaxation in NAS-treated newborns [8,43]. In the case of the infant presented here, we observed a successful decrease in the use of pharmacological medication for sedation with the improvement of the baby’s behaviour through doing Shen Men as part of the NADA protocol. 

Owing to its increased use in children, the safety of acupuncture has been investigated. Adams et al. [44] have reviewed 18 databases, resulting in 37 reports. Of the adverse events reported, the majority were mild in nature, including bruising, and pain. In addition, systematic reviews of acupuncture for children and newborns have found that acupuncture is in fact a safe treatment when performed by trained and licensed acupuncturists [15,44].

In our case, the physicians carrying out the acupuncture were specialists in paediatric and adolescent medicine as well as in neonatology and intensive care medicine; both had a diploma in acupuncture, in NADA, in auricular acupuncture, and in Shonishin, certified by the Austrian Medical Association.

**Figure 2:**
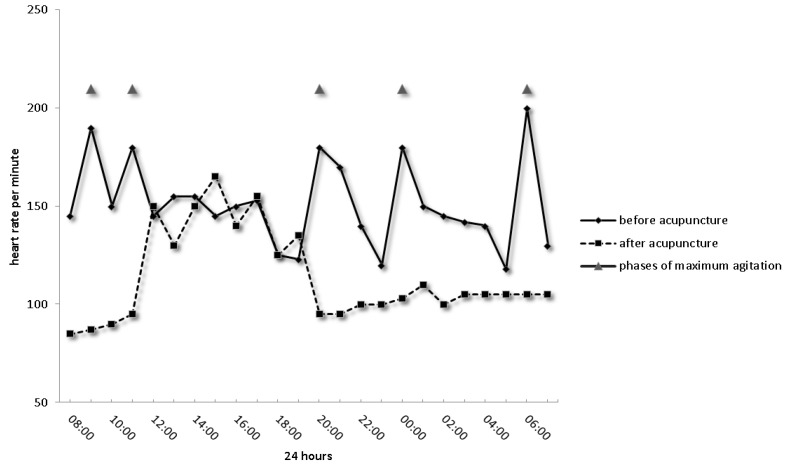
Many phases of pain with elevated heart rate and periods of agitation (grey triangles). After six acupuncture sessions, nearly normal infant behaviour patterns and vital parameters had been established.

Needling Yin Tang and Shen Men as the first and last points, respectively, for anxiolytic and pain reduction resulted in decreased Finnegan Scores on the day of acupuncture treatment and the day after. Furthermore, we could document that the infant fell asleep, body tension improved and heart rate diminished significantly (Illustrated in Figure 2). In combination with LR3 and ST36, drug-therapy was reduced contentiously, bottle feeding established quickly and the nurses’ protocol described a more relaxed baby after the acupuncture treatment.

## 5. Conclusions

In conclusion, it can be said that our case has shown that acupuncture performed by trained acupuncturist paediatricians is a helpful non-pharmacological treatment tool for reducing pain and agitation. While acupuncture is typically used in the outpatient setting, it can also be used in the inpatient setting, as exemplified by the positive outcome in the above case. 

This case report thus adds to the literature on the usefulness of acupuncture in paediatric samples in the hospital setting. 

In addition, we recommend that each acupuncture study protocol be supervised by a trained acupuncturist in accordance with the STRICTA Declaration [19], an extension of the Consolidated Standards of Reporting Studies (CONSORT) statement [18].

## References

[1] Meyer S., Gortner L., Larsen A., Kutschke G., Gottschling S., Gräber S., Schroeder N. (2013). Complementary and alternative medicine in paediatrics: A systematic overview/synthesis of Cochrane Collaboration reviews. Swiss Med. Wkly..

[2] Jindal V., Ge A., Mansky P.J. (2008). Safety and Efficacy of Acupuncture in Children a Review of the Evidence. J. Pediatr. Hematol. Oncol..

[3] Huang T.P., Liu P.H., Lien A.S., Yang S.L., Chang H.H., Yen H.R. (2013). Characteristics of traditional Chinese medicine use in children with asthma: A nationwide population-based study. Allergy.

[4] Thiel M., Stockert K. (2013). Acupuncture in Neonates—Old Experience or New Evidence?. J. Neonatal. Biol..

[5] Kemper K.J., Vohra S., Walls R. (2008). The use of complementary and alternative medicine in pediatrics. Pediatrics.

[6] Rusy L.M., Weisman S.J., Heinsworth K.R. (2013). Developing an in-patient acupuncture treatment in a pediatric hospital. J. Complement. Integr. Med..

[7] Golianu B., Yeh A.M., Brooks M. (2014). Acupuncture for Pediatric Pain. Children.

[8] Gentry K.R., McGinn K.L., Kundu A., Lynn M.A. (2012). Acupuncture therapy for infants: A preliminary report on reasons for consultation, feasibility, and tolerability. Paediatr. Aneasth..

[9] Gottschling S., Reindl T.K., Meyer S., Berrang J., Henze G., Graeber S., Ong M.F., Graf N. (2008). Acupuncture to alleviate chemotherapy-induced nausea and vomiting in pediatric oncology—A randomized multicenter crossover pilot trial. Klin. Paediatr..

[10] Skjeie H., Skonnord T., Fetveit A., Brekke M. (2013). Acupuncture for infantile colic: A blinding-validated, randomized controlled multicentre trial in general practice. Scand. J. Prim. Health Care.

[11] Landgren K., Kvorning N., Hallström I. (2010). Acupuncture reduces crying in infants with infantile colic: A randomised, controlled, blind clinical study. Acupunct. Med..

[12] Reinthal M., Lund I., Ullman D., Lundeberg T. (2011). Gastrointestinal symptoms of infantile colic and their change after light needling of acupuncture: A case series study of 913 infants. Chin. Med..

[13] Landgren K., Kvorning N., Hallström I. (2011). Feeding, stooling and sleeping patterns in infants with colic—A randomized controlled trial of minimal acupuncture. BMC Complement. Altern. Med..

[14] Ecevit A., Ince D.A., Tarcan A., Cabioglu M.T., Kurt A. (2011). Acupuncture in preterm babies during minor painful procedures. J. Tradit. Chin. Med..

[15] Raith W., Urlesberger B., Schmölzer G.M. (2013). Efficacy and safety of acupuncture in preterm and term infants. Evid.-Based. Complement. Alternat. Med..

[16] Chen K.L., Quah-Smith I., Schmölzer G.M., Niemtzow R., Oei J.L. (2017). Acupuncture in the neonatal intensive care unit-using ancient medicine to help today’s babies: A review. J. Perinatol..

[17] Ching S.W., Shu L. (1990). A standard international acupuncture nomenclature: Memorandum from a WHO meeting. Bull. World Health Organ..

[18] MacPherson H., Altman D.G., Hammerschlag R., Lin Y., Wu T., White A., Moher D., STRICTA Revision Group (2010). Revised standards for reporting interventions in clinical trials of acupuncture (STRICTA): Extending the CONSORT statement. Acupunct. Med..

[19] Schulz K.F., Altman D.G., Moher D. (2010). CONSORT 2010 Statement: Updated guidelines for reporting parallel group randomised trials. BMC Med..

[20] Wang S.M., Escalera S., Lin E.C., Maranets I., Kain Z.N. (2008). Extra-1 acupressure for children undergoing anesthesia. Anesth. Analg..

[21] Tugcu A.U., Cabioglu T., Abbasoglu A., Ecevit A., Ince D.A., Tarcan A. (2015). Evaluation of peripheral perfusion in term newborns before and after Yintang (EX-HN 3) massage. J. Tradit. Chin. Med..

[22] Kim M.S., Seo K.M. (2007). Effects of Atipamezole and Naloxone on Electroencephalographic Spectral Edge Frequency 95 in Dogs Sedated by Acupuncture at GV20 and Yintang Point. J. Vet. Med. Sci..

[23] Wu C., Qu S., Zhang J., Chen J., Zhang S., Li Z., Chen J., Ouyang H., Huang Y., Tang C. (2014). Correlation between the Effects of Acupuncture at Taichong (LR3) and Functional Brain Areas: A Resting-State Functional Magnetic Resonance Imaging Study Using True versus Sham Acupunture. Evid. Based Complement. Altern. Med..

[24] Wu S., Sapru A., Stewart M.A., Milet M.J., Hudes M., Livermore L.F., Flori H.R. (2009). Using acupuncture for acute pain in hospitalized children. Pediatr. Crit. Care Med..

[25] Usichenko T.I., Wolters P., Anders E.F., Splieth C. (2016). Acupuncture Reduces Pain and Autonomic Distress during Injection of Local Anesthetic in Children: A Pragmatic Crossover Investigation. Clin. J. Pain.

[26] Lin Y.C., Tassone R.F., Jahng S., Rahbar R., Holzman R.S., Zurakowski D., Sethna N.F. (2009). Acupuncture management of pain and emergence agitation in children after bilateral myringotomy and tympanostomy tube insertion. Paediatr. Anaesth..

[27] Lu G.W. (1983). Characteristics of afferent fiber innervation on acupuncture points zusanli. Am. J. Physiol..

[28] Chang C.S., Ko C.W., Wu C.Y., Chen G.H. (2001). Effect of Electrical Stimulation on Acupuncture Points in Diabetic Patients with Gastric Dysrhythmia: A Pilot Study. Digestion.

[29] Tsai S.L., Fox L.M., Murakami M., Tsung J.W. (2016). Auricular Acupuncture in Emergency Department Treatment of Acute Pain. Ann. Emerg. Med..

[30] Capone F., Assenza G., Di Pino G., Musumeci G., Ranieri F., Florio L., Barbato C., Di Lazzaro V. (2015). The effect of transcutaneous vagus nerve stimulation on cortical excitability. J. Neural Transm..

[31] World Health Organization (2002). Acupuncture: Review and Analysis of Reports on Controlled Clinical Trials.

[32] Wong V., Cheuk D.K., Chu V. (2013). Acupuncture for hypoxic ischemic encephalopathy in neonates. Cochrane Database Syst. Rev..

[33] Carbajal R., Rousset A., Danan C., Coquery S., Nolent P., Ducrocq S., Saizou C., Lapillonne A., Granier M., Durand P. (2008). Epidemiology and treatment of painful procedures in neonates in intensive care units. JAMA.

[34] Kundu A., Jimenez N., Lynn A. (2008). Acupuncture therapy for prevention of emergence delirium in children undergoing general anesthesia. Med. Acupunct..

[35] Hsiu H., Hsu W.C., Hsu C.L., Huang S.M. (2011). Assessing the effects of acupuncture by comparing needling the hegu acupoint and needling nearby nonacupoints by spectral analysis of microcirculatory laser Doppler signals. Evid. Based Complement. Altern. Med..

[36] Min S., Lee H., Kim S.Y., Park J.Y., Chae Y., Lee H., Park H.J. (2015). Local Changes in Microcirculation and the Analgesic Effects of Acupuncture: A Laser Doppler Perfusion Imaging Study. J. Altern. Complement. Med..

[37] Yates C.C., Mitchell A.J., Lowe L.M., Lee A., Hall R.W. (2013). Safety of Noninvasive Electrical Stimulation of Acupuncture Points during a Routine Neonatal Heel Stick. Med. Acupunct..

[38] Chung J.W., Yan V.C., Zhang H. (2014). Effect of acupuncture on heart rate variability: A systematic review. Evid.-Based Complement. Altern. Med..

[39] He W., Rong P.J., Li L., Ben H., Zhu B., Litscher G. (2012). Auricular acupuncture may suppress epileptic seizures via activating the parasympathetic nervous system: A hypothesis based on innovative methods. Evid.-Based Complement. Altern. Med..

[40] Cotton S., Luberto C.M., Bogenschutz L.H., Pelly T.H., Dusek J. (2014). Integrative Care Therapies and Pain in Hospitalized Children and Adolescents: A Retrospective Database Review. J. Altern. Complement. Med..

[41] Raith W., Schmölzer G.M., Resch B., Reiterer F., Avian A., Koestenberger M., Urlesberger B. (2015). Laser Acupuncture for Neonatal Abstinence Syndrome: A Randomized Controlled Trial. Pediatrics.

[42] Schwartz L., Xiao R., Brown E.R., Sommers E. (2011). Auricular acupressure augmentation of standard medical management of the neonatal Narcotic Abstinence Syndrome. Med. Acupunct..

[43] Raith W., Litscher G., Müller W., Urlesberger B. (2013). Laseracupuncture—A possible alternative treatment for agitation and pain in neonates?. Paediatr. Anaesth..

[44] Adams D., Cheng F., Jou H., Aung S., Yasui Y., Vohra S. (2011). The safety of pediatric acupuncture: A systematic review. Pediatrics.

